# Identifying Residues for Substrate Recognition in Human GPAT4 by Molecular Dynamics Simulations

**DOI:** 10.3390/ijms25073729

**Published:** 2024-03-27

**Authors:** Yulan Liu, Yunong Xu, Yinuo Xu, Zhihao Zhao, Gui-Juan Cheng, Ruobing Ren, Ying-Chih Chiang

**Affiliations:** 1 School of Medicine, The Chinese University of Hong Kong, Shenzhen 518172, China; 2 Kobilka Institute of Innovative Drug Discovery, School of Medicine, The Chinese University of Hong Kong, Shenzhen 518172, China; 3 Warshel Institute for Computational Biology, School of Medicine, The Chinese University of Hong Kong, Shenzhen 518172, China; 4 Shanghai Key Laboratory of Metabolic Remodeling and Health, Institute of Metabolism and Integrative Biology, Fudan University, Shanghai 200438, China; 5 Shanghai Qi Zhi Institute, Shanghai 200232, China

**Keywords:** glycerol-3-phosphate acyltransferase, triacylglycerol synthesis, AlphaFold structure

## Abstract

Glycerol-3-phosphate acyltransferase (GPAT) catalyzes the first step in triacylglycerol synthesis. Understanding its substrate recognition mechanism may help to design drugs to regulate the production of glycerol lipids in cells. In this work, we investigate how the native substrate, glycerol-3-phosphate (G3P), and palmitoyl-coenzyme A (CoA) bind to the human GPAT isoform GPAT4 via molecular dynamics simulations (MD). As no experimentally resolved GPAT4 structure is available, the AlphaFold model is employed to construct the GPAT4–substrate complex model. Using another isoform, GPAT1, we demonstrate that once the ligand binding is properly addressed, the AlphaFold complex model can deliver similar results to the experimentally resolved structure in MD simulations. Following the validated protocol of complex construction, we perform MD simulations using the GPAT4–substrate complex. Our simulations reveal that R427 is an important residue in recognizing G3P via a stable salt bridge, but its motion can bring the ligand to different binding hotspots on GPAT4. Such high flexibility can be attributed to the flexible region that exists only on GPAT4 and not on GPAT1. Our study reveals the substrate recognition mechanism of GPAT4 and hence paves the way towards designing GPAT4 inhibitors.

## 1. Introduction

Triacylglycerol (TAG) is a common glycerol lipid. It is the major constituent of body fat in humans and therefore its synthesis in cells has attracted great attention. In cells, TAG synthesis begins with attaching an acyl group to the *sn*-1 position of glycerol-3-phosphate (G3P), forming lysophosphatidic acid (LPA). This reaction is catalyzed by an enzyme termed glycerol-3-phosphate acyltransferase (GPAT) [1,2], which has four isoforms, GPAT1, GPAT2, GPAT3 and GPAT4, in humans. GPAT1 and GPAT2 are located in the outer mitochondrial membrane. The former catalyzes the first step in TAG synthesis and is highly expressed in the liver and other tissues with a high capacity for TAG synthesis [3]. It accounts for about 30% to 50% of GPAT activity [4] in the liver and has been proposed to be a compelling target for nonalcoholic fatty liver disease [5]. GPAT3 and GPAT4 are found on the endoplasmic reticulum membrane [1], and the latter also accounts for about 50% of the GPAT activity in the liver [6,7]. Additionally, in a study, GPAT4-deficient mice suffered from subdermal lipodystrophy and were resistant to obesity induced by a high-fat diet [7,8]. Hence, knowledge of how GPAT4 binds with its native substrate is of great interest for the purpose of drug design. Early studies have applied multiple sequence alignment in order to understand the substrate binding process. Upon aligning the sequences of human GPAT1 with the sequences of other acyltransferases from bacteria, yeast, nematodes and mammals, Lewin et al. discovered four blocks with many highly conserved residues [9], which were later identified as four motifs in the literature [7]. The functions of these motifs were further “verified” via site-directed mutagenesis experiments [9], providing limited insights into the substrate binding in the first step of TAG synthesis. Apart from these four motifs, GPAT4 has a fifth motif, which is required to achieve its enzymatic activity [10].

Substrate binding information can be best determined by resolving the enzyme–substrate complex structure, either by X-ray crystallography or by cryo-electron microscopy (cryo-EM). Unfortunately, only a few GPAT structures have been resolved so far, including two structures from squash [11,12], six structures from *E. coli* [13] and two structures from human GPAT1 (PDB codes 8E4Y and 8E50) [5]. Traditionally, in order to investigate the GPAT4–substrate binding in such a scenario, where no experimentally resolved structure is available, one would carry out homology modeling [14], which requires a certain level of sequence similarity between the target sequence (GPAT4 here) and the template sequence (GPAT1 here). Nowadays, due to advances in machine learning in recent years, protein structures can be predicted accurately [15], and a dedicated database is also available for all protein sequences [16].

A question naturally arises: Can we investigate the GPAT4–G3P–CoA binding using computational approaches, using a model structure predicted by AlphaFold2? While one may question the accuracy of its predictions, AlphaFold2 has already achieved median backbone accuracy of 0.96 Å RMSD_95_ (C_α_ RMSD deviation at 95% residue coverage) and all-atom accuracy of 1.5 Å RMSD_95_ for 87 protein domains in the CASP14 competition [15]. He et al. also compared the structures of 29 G-protein-coupled receptors obtained from cryo-EM with predictions from AlphaFold2. The associated C_α_ RMSD showed an average deviation of 1.64±1.08 Å, indicating strong alignment between the AlphaFold2 predictions and experimental data [17]. A recent GPAT1 structural investigation confirmed that AlphaFold2’s prediction of GPAT1 (termed GPAT1’s AlphaFold model hereafter) agreed well with the experimentally resolved GPAT1 structure [5]: the overall root-mean-square deviation (RMSD) of C_α_ was merely 1.45 Å. This suggests that the GPAT4 structure taken from the AlphaFold database may be a good approximation of the actual structure. Furthermore, if a reliable complex model of GPAT4–G3P–CoA can be constructed, then one can investigate the ligand binding using molecular dynamics (MD) simulations.

In this work, we first compare the structures of GPAT1 and GPAT4. After identifying the similarity between their pockets, we then discuss how a reliable complex model can be constructed using the GPAT1 structure taken from the AlphaFold protein structure database [16] (AF-Q9HCL2-F1-model_v4) and structures of ligands from PDB (code 8E50). We choose 8E50 because it reflects the structure of GPAT1 and its products, which allows us to model the reactants for our study. The best protocol learned from the GPAT1 study is then employed to construct the GPAT4–G3P–CoA complex, using the associated model (AF-Q86UL3-F1-model_v4) taken from the same database. MD simulations are employed to reveal G3P’s recognition mechanism in GPAT4, and the results are compared with those of GPAT1 to highlight the differences between the two enzyme isoforms.

## 2. Results and Discussion

### 2.1. Multiple Sequence Alignment and Structural Analysis

In order to identify the crucial residues for the recognition of G3P, we use Clustal Omega [18] to align the sequences of the four human GPAT isoforms with other acyltransferases reported in the literature [9].

**Figure 1:**
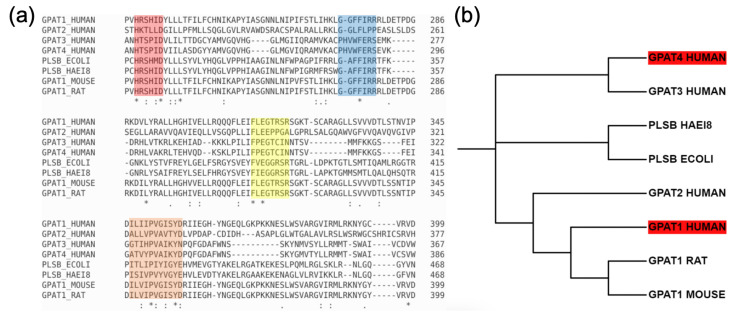
(**a**) Result of multiple sequence alignment. Listed sequences are Human GPAT1 to Human GPAT4, GPAT (PlsB) from *Escherichia coli*, GPAT (PlsB) from *Haemophilus influenzae*, GPAT1 from mouse and GPAT1 from rat. Motifs I, II, III, IV are highlighted in red, blue, yellow and orange, respectively. Notably, conserved residues (indicated by *) and conservative replacements (indicated by :) are clustered around motif I and motif IV. The semi-conservative replacements (indicated by .) are also labeled. (**b**) The phylogenetic tree of the aligned sequences shows their phylogenetic relatedness. Despite having evolved from a common ancestor, GPAT4 shares limited relatedness with GPAT1 and GPAT2 compared to GPAT3. The phylogenetic tree was constructed using the Maximum Likelihood method.

The result is depicted in Figure 1a. The sequences of the four reported motifs (I, II, III, IV) are highlighted in red, blue, yellow and orange, respectively. According to an earlier report [7], motifs I and IV are associated with catalysis, while motifs II and III are associated with G3P binding. However, a more recent report on the structure of GPAT1 showed that motif IV (X)_4_P(X)_3_ is associated with the structure’s stability instead of catalysis [5]. The same article also pointed out that motif II is responsible for binding with palmitoyl-Coenzyme A (CoA) instead of G3P. Only the functions of motif I (HXXXXD) and motif III are in line with the earlier prediction, viz. motif I for catalysis and motif III for G3P binding. However, in our sequence alignment with GPAT4, conserved residues (indicated by *) and conserved replacements (indicated by :) only appear in clusters around motif I and motif IV, and not around motif II and motif III. This result differs from those of an earlier study [9], where the GPAT3 and GPAT4 sequences were not aligned. The lack of conserved residues around motifs II and III implies that these sequences on GPAT4 may have different functions in comparison with those on GPAT1. In particular, GPAT4 may use residues other than motif III to recognize G3P.

The phylogenetic tree (Figure 1b) shows that GPAT1 and GPAT4 are not so closely related. The difference in their sequence length reflects this too: GPAT1 has 828 amino acids, but GPAT4 has only 456 amino acids. This results in very low sequence similarity (17.53%). Consequently, the homology model of GPAT4 built from the GPAT1 structure is rather poor. Either the four motifs appear in the incorrect places or the model has many unstructured loops. In contrast, the AlphaFold models of GPAT1 and GPAT4 show good confidence, with a predicted local distance difference test (pLDDT) ≥ 70 for the overall protein structure, and high confidence (pLDDT ≥ 90) around the binding pocket. Only some loops exhibit lower confidence. Since the AlphaFold prediction for GPAT1 has been proven to be quite accurate when compared with experiments, the GPAT4 structure with similar quality in prediction should also be reliable. The structures of the simple homology models and the quality assessment of the AlphaFold models are provided in the Supplementary Information (SI) in Figure S1 and Figure S2, respectively.

**Figure 2:**
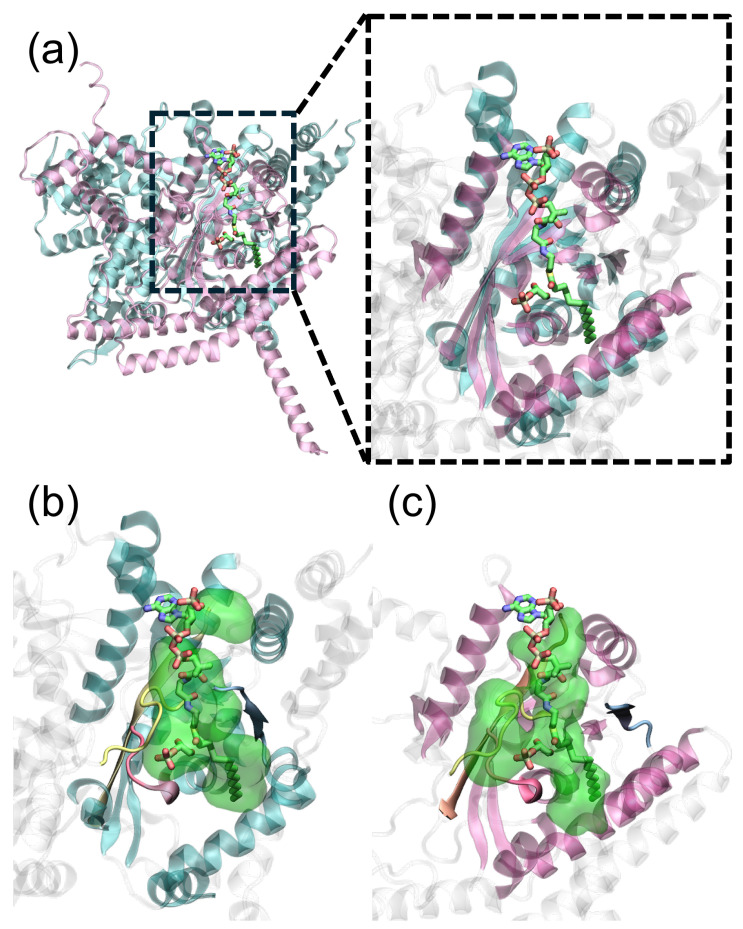
Binding pockets of GPAT1 and GPAT4. (**a**) Structure alignment of GPAT1 (8E50, cyan) and GPAT4 (AlphaFold, mauve). The two proteins have a very similar binding pocket, as indicated by the associated secondary structures. (**b**) The binding pocket of GPAT1 (8E50) found by MOE-SiteFinder. The pocket depicted by the green transparent volume is a combination of one large cavity and five adjacent small cavities. Motifs I to IV are colored in red, blue, yellow and orange, respectively. Ligands are colored in green. (**c**) The largest binding pocket of GPAT4 (AlphaFold) identified by MOE-SiteFinder (green transparent volume). It coincides with GPAT1’s binding site, which is indicated by the ligands (green).

Upon aligning the protein structures, one finds that the binding pockets of GPAT1 and of GPAT4 are quite similar. This is reflected in the close overlap between the protein secondary structures of the binding pocket, cf. the mauve and cyan structures shown in Figure 2a. Binding site identification also suggests that the pockets of the two proteins overlap in space: shown in Figure 2b,c are the cavities identified by MOE-SiteFinder [19]. It employs spheres to probe voids in proteins and reports the cavity of a potential binding pocket as well as the residues around the pocket. Three large cavities as well as multiple small cavities are identified on the GPAT1 structure (8E50). The third-largest one and five small ones form the complete binding site known from the experiment. On the other hand, the largest cavity found on GPAT4 coincides with GPAT1’s binding pocket, which is indicated by the reference ligands (green).

**Figure 3:**
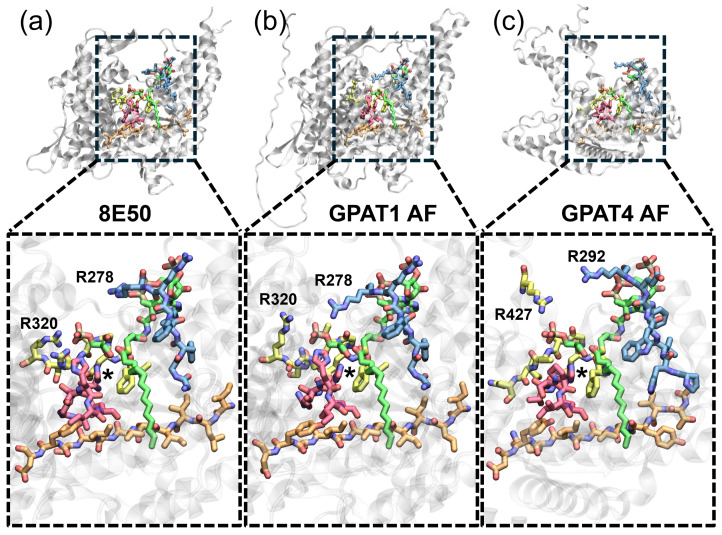
Structures of motifs I to IV. In order, they are colored in red, blue, yellow and orange, respectively. Ligands are colored in green. The catalytic histidine is labeled by *. (**a**) GPAT1 structure taken from 8E50. Residue R320 clearly interacts with the phosphate group on G3P. (**b**) GPAT1 structure taken from the AlphaFold model. G3P and CoA are depicted for reference. The RMSD of the four motifs’ heavy atoms is 2.14 Å between 8E50 and the current model. Notably, R278 should interact with CoA as in 8E50, but it now appears within 4 Å of G3P. (**c**) GPAT4 structure taken from the AlphaFold model. G3P and CoA are depicted for reference. Unlike GPAT1, there is no arginine on motif III (yellow) to interact with G3P. The nearby ARG427 is not located on motif III.

Furthermore, upon structural alignment, the four motifs naturally appear at the same locations, confirming the fact that the two enzymes catalyze the same function. Figure 3a–c depict the structure of the four motifs in 8E50, the GPAT1 AlphaFold model and the GPAT4 AlphaFold model, respectively. Substrates G3P and CoA are also plotted for reference. R320 in the AlphaFold model (Figure 3b) points away from the reference G3P, but the same residue forms a stable salt bridge with the phosphate group on G3P in 8E50 (Figure 3a). This supports the role of motif III (yellow) in G3P’s binding. Motif I (red) on GPAT1 is found close to G3P, supporting the proposed catalytic mechanism. In contrast to GPAT1, GPAT4 has no arginine on motif III to recognize G3P. This raises a question regarding how GPAT4 can recognize G3P without positively charged residues on the motif. Finally, the extra motif V of GPAT4 is located on the β-sheet next to motif IV. Judging from its location, this motif is unlikely to participate in the catalytic reaction but is more likely to maintain the structure’s stability, like motif IV; see Figure S3 for motif V on GPAT4.

### 2.2. GPAT1 Molecular Dynamics Simulations: Complex Built with AlphaFold Model and Experimentally Resolved Complex Yield Similar Results

To address the question of how G3P is recognized by GPAT4, a reliable GPAT4–G3P–CoA complex must be constructed first. Thus, our next goal is to find out how to construct a reliable enzyme–substrate complex using the AlphaFold model. To do so, we compare the results of MD simulations performed with three different GPAT1–G3P–CoA complexes. The first complex (labeled as 8E50) is built from the PDB structure, as depicted in Figure 3a. While the original PDB file provides only the structures of the products, i.e., LPA and CoA without the acyl group, the reactant ligands (G3P and palmitoyl-CoA) can be modeled by linking the acyl group to CoA instead of G3P, followed by energy minimization. This can be done using the Molefacture plugin in VMD [20], resulting in reactants with nearly identical atomic positions to those in 8E50. The corresponding RMSD is only 0.5 Å; see Figure S4. The second complex (labeled as AF+ligands) is built using the GPAT1 AlphaFold model (AF-Q9HCL2-F1-model_v4) with the ligands taken from 8E50, i.e., reactants modeled following the protocol described previously. The complex is then subjected to energy minimization with all heavy atoms on the complex constrained, in order to correct the steric clashes between the inserted ligands and the protein residues, while maintaining the original complex conformation. The energy minimization removes the steric clashes between CoA and S255, G256 and Y236 that block the passage to the binding pocket of the acyl group. Additionally, it also removes the clashes of L280, L271, L237 and L193 with CoA. See Figure S5 for the conformations before and after the constrained energy minimization. The last complex (labeled as AF+docking) employs the same AlphaFold model but now the ligand binding poses are determined by a naive docking protocol, where the protein conformation is unchanged (rigid docking). As shown in Figure S4, the docking pose differs greatly from the ligand pose modeled from PDB, with a large RMSD of 9.91 Å. Such a large deviation is mainly caused by the poor conformation of the protein residues, particularly Y236, S255 and G256. These three residues block the passage to the non-polar pocket. This inappropriate residue conformation leads to extremely poor CoA docking poses, with the acyl group binding at the incorrect place, as shown in Figure S6a–c. Such performance is not surprising, as it has been reported in the literature that AlphaFold structures may not be a good choice for docking or virtual screening [21,22]. As a reference, docking CoA back to the 8E50 GPAT1 structure yields similar poses to the CoA pose modeled from 8E50; see Figure S6d–f.

After the construction of the initial complex, CHARMM-GUI [23] is employed to construct the solvated complex–membrane system for simulation. Notably, GPAT1 is not a transmembrane protein but a monotopic membrane protein, as suggested by its structure [5]. GPAT4 is a bitopic protein that is anchored on the membrane surface by a hydrophobic helix. Both proteins are mostly exposed to the solvent. Figure S7 shows how GPAT1 and GPAT4 are integrated onto the membrane. Other details can be found in the Materials and Methods.

**Figure 4:**
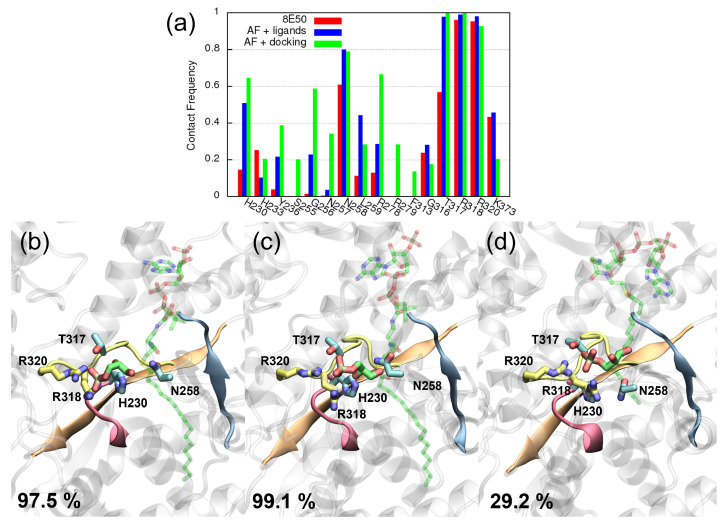
Results of the MD simulations using three different complex models: the complex modeled from PDB (8E50), the complex modeled from AlphaFold with ligands from PDB (AF+ligands) and the complex from AlphaFold with ligands from molecular docking (AF+docking). (**a**) The contact frequency with G3P obtained from MD trajectories. Important residues such as R318 and R320 have a high contact frequency over all three complex models. These two residues recognize the native substrate through the electrostatic interaction with the phosphate group on G3P. Panels (**b**–**d**) depict the first cluster centroid found in the MD simulations of model 8E50, model AF+ligands and model AF+docking, respectively. The former two have a more dominant cluster, viz. 97.5% and 99.1% of the trajectory length. MD simulations find no dominant ligand conformation in model AF+docking, where the largest cluster size is merely 29.2% of the trajectory. G3P is colored in green. CoA is shown in translucent green. Positively charged residues within 3 Å of G3P’s phosphate group are shown in yellow, while T317, N258 and H230 are colored in cyan. Residues R278 and K373 also appear within 3 Å of G3P in model AF+docking. For clearer visualization, these two residues are depicted in Figure S9.

For each system, three independent 500 ns MD simulations are conducted and combined for analysis. As shown in Figure 4a, R318 and R320 on motif III have the highest contact frequency with G3P across all three complex models, indicating that a stable interaction is formed between the two residues and G3P. Second, the catalytic H230 also interacts with G3P, but its contact frequency is much lower in the 8E50 model (red) than in the other two models. Although the contact frequency of H230 is not high, e.g., only 14.5% for the 8E50 model, the reaction can still take place. Based on the results from the 8E50 model, other residues that frequently appear within 3 Å of G3P include H233, N258, G316, T317 and K373. They may form hydrogen bonds with G3P or interact with it via electrostatic interaction, but these interactions are transient compared to the charge–charge interaction between R318/R320 and G3P.

The AF+ligands model (blue) also finds the same residues in contact with G3P, but some of them show a higher contact frequency than 8E50. For example, the catalytic H230 shows a much higher contact frequency in the AF+ligands model, and so do L259, R278 and T317. A similar trend, viz. that the protein has more contact with G3P, is also observed in simulations of the AF+docking model (green). One can even notice some residues, such as N257 or R279, having a high contact frequency with G3P in the simulations of the AF+docking model, but these residues have no contact with G3P in the simulations of the 8E50 model. Upon comparison, we conclude that the AF+ligands model better reproduces the 8E50 model in terms of the contact frequency calculated from the associated MD simulations, cf. the red and blue bars in panel (a). As for the AF+docking model, although it finds many more false contacts, it still provides some important features: R318 and R320 participate in substrate recognition, and other important residues such as H230 are still in contact with the substrate.

We further compare the major ligand poses found by the MD simulations. Shown in Figure 4b–d are the conformations of the major cluster centroid of G3P and CoA, obtained from the simulations of model 8E50, model AF+ligands and model AF+docking, respectively. These clusters are obtained using an RMSD cutoff of 3 Å on the ligands (both CoA and G3P), while the protein is aligned. Consequently, each cluster contains only frames with similar ligand binding poses, and hence the clusters can be used to identify different ligand binding modes. Including the residues with a high contact frequency in the clustering protocol will not affect the major binding modes significantly; see Figure S8. In panel (b) and (c), G3P’s orientation is slightly different, but the interactions with R320 and with R318 are kept, as is CoA’s pose. Overall, the two binding poses shown in panels (b) and (c) are still rather similar. In contrast, panel (d) shows a huge difference in CoA’s binding pose. This major cluster only represents 29.2% of the associated trajectories, i.e., no dominant binding pose is found. Conversely, for models 8E50 and AF+ligands, the major cluster represents 97.5% and 99.1% of the simulations, respectively. This much lower cluster size in AF+docking originates from the fluctuation in CoA during the simulations. This is expected because the acyl group fails to insert into the non-polar pocket during the rigid docking. If the cluster analysis was performed over only the G3P trajectory (ignoring CoA), the major cluster size would be 77.4%.

### 2.3. GPAT4 Molecular Dynamics Simulations Reveal G3P’s Recognition Mechanism

Based on the structural similarity of the binding pocket between GPAT1 and GPAT4, the identical location of the catalytic residues (motif I) in space and the same enzymatic function, we assume that ligands bind in a similar pose in both GPAT1 and GPAT4. This assumption is supported by the distribution of positively charged and hydrophobic residues over the protein. Following the protocol validated above, we construct the GPAT4 complex using the model taken from the AlphaFold protein structure database (AF-Q86UL3-F1-model_v4) with the ligands from PDB 8E50. The constructed system is then subjected to 500 ns MD simulations. In total, eight independent copies of simulations are performed.

**Figure 5:**
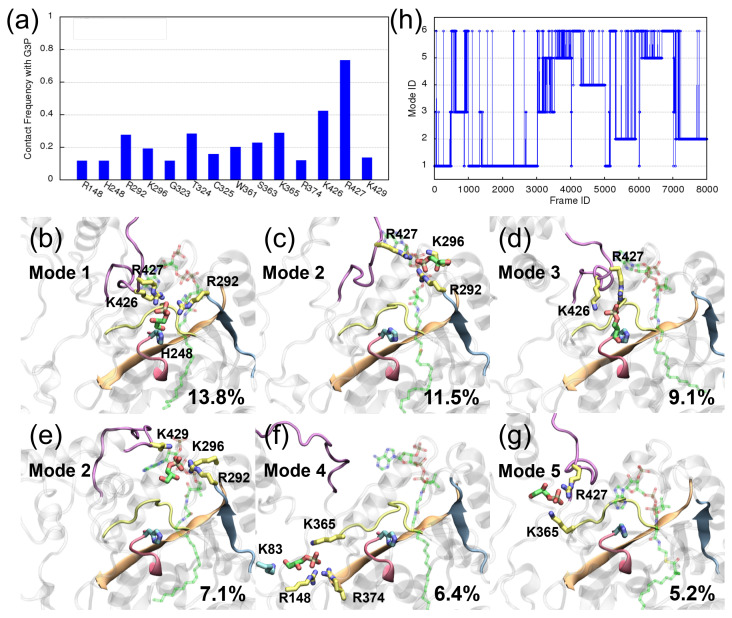
Results of MD simulations for the GPAT4 complex. (**a**) The contact frequency with G3P. R427 is the major residue interacting with G3P in all trajectories. Panels (**b**–**g**) depict the ligands’ centroid conformations of the first to the sixth cluster obtained from the simulations, respectively. The corresponding cluster sizes are 13.8%, 11.5%, 9.1%, 7.1%, 6.4%, 5.2% of the trajectory length, respectively. G3P is again colored in green. CoA is colored in translucent green. Positively charged residues within 3 Å of G3P’s phosphate group are colored in yellow. Other residues are colored in cyan. Residues 419 to 431 (loop in purple) show a large fluctuation over these clusters. (**h**) Transition between different binding modes over the simulations. Binding modes are assigned according to the electrostatic interactions observed in panels (**b**–**g**). See the text for details.

Different from what is observed in the GPAT1 complex simulations, the substrate binding seems to be less stable here: as shown in Figure 5, R427 on GPAT4 is responsible for recognizing G3P, but its contact frequency with G3P comprises only about 80% of the trajectories, whereas the corresponding residues on GPAT1 (R320 and R318) are always in contact with G3P during the simulations. Second, the residues with a high contact frequency on GPAT1 are located around G3P’s binding pocket, while, in GPAT4, many arginines and lysines away from the binding pocket also show a significant interaction with G3P. This suggests that G3P wanders away from the binding pocket in some of the GPAT4 simulations. Indeed, as shown below, G3P leaves GPAT4’s binding pocket in five of the eight simulations.

The centroids of the first six clusters of ligands obtained from the GPAT4 complex trajectories are depicted in Figure 5b–g. As depicted in the panels, G3P shows a large fluctuation in space, along with the large fluctuation of R427 caused by the loop motion; see the purple loop depicted in the panels. The fluctuation of G3P is also reflected in the sizes of these six clusters, which account for 13.8%, 11.5%, 9.1%, 7.1%, 6.4% and 5.2% of the trajectory length. These centroids reveal the existence of several potential traps formed by lysines and arginines. For instance, in panel (b), G3P interacts with R427, R292 and K426 via electrostatic interactions and with H248 via hydrogen bonds. In other words, R427, R292 and K426 form a potential trap to attract G3P. Figure 5c,e show the same pattern of residue interactions: R427, K296 and R292 form another potential trap to attract G3P. The same is shown for R427 and K426 in panel (d); K365, R148 and R374 in panel (f); and R427 and K365 in panel (g).

The charged residues revealed in Figure 5 can form stable interactions with G3P’s phosphate group, leading to stable interatomic distances that can be used to separate different interaction patterns between G3P and the protein residues, viz. binding modes that are related to certain pairs of protein–ligand interactions. For example, take the sixth cluster shown in panel (g): its G3P interacts with R427 and K365, so the distances from G3P’s phosphorus atom to R427’s CZ atom and to K365’s NZ atom are constantly below 5 Å. Hence, the associated distances can be used to identify this mode (mode 5). Similarly, the distances between G3P’s phosphorus atom and K365’s NZ atom, R148’s CZ and R374’s CZ atom can be used to characterize mode 4, which is depicted in Figure 5f. However, as the interaction between G3P and R148/R374 is unique to this mode, it is sufficient to use only these two distances to identify mode 4. Different from the simple one-to-one mapping between the cluster and the binding pattern, sometimes, two clusters may have similar binding patterns and could be considered as the same mode. For instance, G3P binds near the entrance of the binding pocket both in the second and the fourth clusters (Figure 5c,e), with a common interaction of G3P-K296 and G3P-R292 in the two clusters. This allows us to define mode 2 using the associated distances. Finally, we would like to characterize G3P’s binding at the active site (mode 1) and separate those that also interact with R427 but are no longer located at the binding site and do not belong to any of the other modes. The latter often occurs during the transition between different modes and will be termed mode 3. When G3P binds at the active site for reaction, it is physically close to the catalytic H248. It is natural to utilize the distance between G3P and H248 to decide whether G3P locates at the active site. The distance between G3P’s phosphorus atom and H248’s NE atom is employed here, since a steady distance (around 10 Å) can be observed when G3P binds at the active site; see Figure S10 for the interatomic distance. This distance naturally separates mode 1 from the other modes. For the remaining trajectories, those whose G3P interacts with R427 and K426 will be identified as mode 3, and the rest will be assigned as a dummy mode 6 for simplicity. Overall, modes 1, 2, 3, 4 and 5 represent a binding pattern similar to what is shown in panels (b), (c)/(e), (d), (f) and (g), respectively. A summary of the distances used to define each mode is given in Table 1, along with the size of each mode. Because the binding mode is defined purely based on G3P’s interaction pattern, its size is different from the size of the cluster shown in Figure 5.

Upon assigning the binding modes to each frame, one can observe how G3P switches its interactions between different modes, which are associated with different binding hotspots. The eight GPAT4 trajectories are concatenated, and the results are depicted in Figure 5h. Each trajectory is represented using 1000 frames, and the vertical lines connecting different modes represent G3P’s transitions between different hotspots. Clearly, mode 1 appears mainly in the first three trajectories. This agrees with our observation that G3P wanders away in five of the eight simulations, and only three simulations find relatively stable binding of G3P. See the SI for animations of the trajectories. Interestingly, within the first three simulations, a transition between mode 1 and mode 3 is observed. In the latter five simulations, transitions between modes occur even more frequently: a transition from mode 1 to mode 5 via mode 3 is observed from frame ID 3000 to 4000 and frame ID 6000 to 7000. Similarly, a transition from mode 1 to mode 4 via mode 3 is observed from frame ID 4000 to 5000. From frame ID 5000 to 6000, we see the transition from mode 1 to mode 2 via mode 3. Finally, a transition from mode 1 to mode 2 via mode 3 and mode 5 is observed in the last trajectory (frame ID 7000 to 8000).

In comparison with the GPAT1 simulations, G3P wanders between several binding hotspots on GPAT4. Although the exact biological significance of having multiple hotspots on GPAT4 is unclear, in other proteins, this is known to modulate the binding affinity or substrate specificity [24], or even to result in allosteric regulation [25]. G3P’s transition between multiple binding hotspots on GPAT4 can be attributed to a dynamic region, viz. residues 69 to 158 and residues 421 to 456, which are shown in red in Figure S11a. The RMSD depicted in Figure S11c reveals that this region undergoes large-magnitude motion during the MD simulations. This high flexibility does not imply that AF’s prediction is poor for this region. On the contrary, Figure S2 already shows that the predictions of residues 99–86, 108–148 and 419–447 are either highly confident or confident, as is the one of R427. However, as the structural model suggests that GPAT4 is a bitopic membrane protein with most of its surface exposed to the solvent, the less compact region on GPAT4 can undergo large motion, regardless of the confidence level of the prediction. Importantly, the confidently predicted residue R427 is located on this dynamic region, and thus it can bring G3P around to different binding hotspots via its interaction with the phosphate group on the ligand. In contrast, GPAT1’s structure is compact, and hence it is stable when being exposed to the solvent.

While the current simulations reveal an extremely flexible region on GPAT4 (residues 69–158 and 421–456), this region may be stabilized if it can interact with other proteins or with the membrane. Stabilizing this region will likely reduce the magnitude of R427’s motion and consequently stabilize the binding with G3P. In this case, mode 1, shown in Figure 5b, should still provide useful information, as it corresponds to the trajectories where ligands are stably bound inside the pocket.

### 2.4. Features of CoA’s Binding Revealed by Molecular Dynamics Simulations

**Figure 6:**
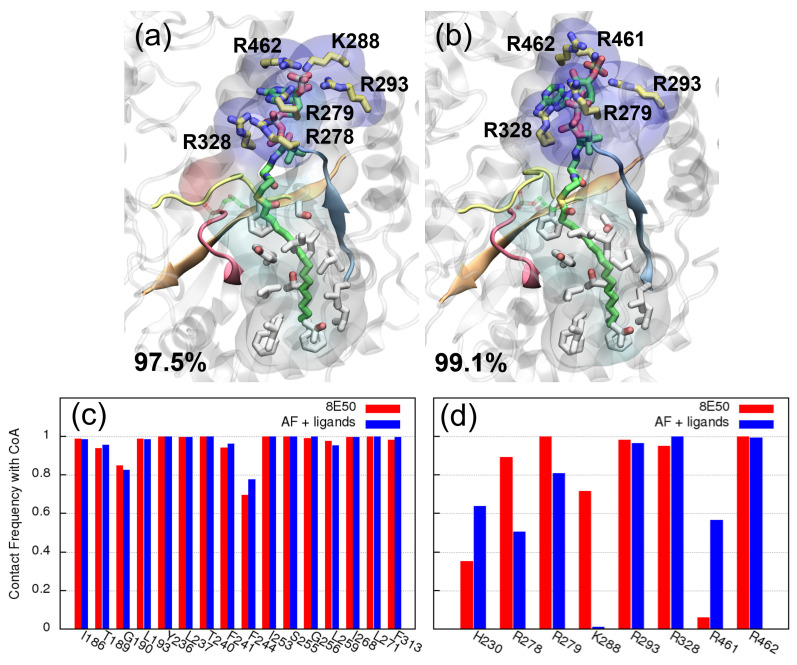
CoA’s binding with GPAT1. (**a**) The centroid of the major cluster found in GPAT1’s MD simulations using the PDB structure (8E50). Positively charged residues within 3 Å of CoA’s phosphate groups are colored in yellow. Residues within 3 Å of CoA’s acyl group are colored in white. The transparent volume depicts all protein residues within 3 Å of CoA, and the color represents the type of residue. Positively charged, negatively charged, hydrophobic and polar residues are colored in blue, red, white and cyan, respectively. The size of the cluster is given as the percentage of the total trajectory length. (**b**) The centroid of the major cluster found in GPAT1’s AF+ligands simulations. (**c**) The contact frequency of residues from the non-polar pocket with CoA. These residues appear within 3 Å of CoA throughout almost the entire simulation, except for G190 and F244. (**d**) The contact frequencies of selected residues with CoA. Selected are those that interact with CoA’s phosphate groups and the catalytic H230. The MD simulations find similar contact frequencies with CoA for both systems (8E50 and AF+ligands), except for K288 and R461. CoA is also frequently in contact with the catalytic H230.

Lastly, we discuss the features of CoA’s binding using the same clusters as before, i.e., clusters built with both G3P and CoA. Since the initial CoA conformation for MD simulations is incorrect in the AF+docking system, the discussion will focus on GPAT1 8E50, GAPT1 AF+ligands and GPAT4 systems. Shown in Figure 6a,b are the major CoA binding poses found in the GPAT1 MD simulations using the PDB (8E50) and the AlphaFold model (AF+ligands), respectively. These are the same centroids as those depicted in Figure 4, but now with a focus on CoA’s binding. As expected, the acyl group of CoA binds at a non-polar pocket (white transparent volume) that is composed mostly of hydrophobic residues in both sets of simulations. The contact frequency between CoA and these residues is in general above 90% of the simulation, indicating stable binding with the acyl group. See Figure 6c for the associated contact frequencies. Apart from this characteristic non-polar binding pocket, GPAT1 interacts with CoA’s phosphate groups via several positively charged residues. This involves multiple arginines and lysines, e.g., R279, R293, R328, R462. These residues locate around the pocket entrance and show a high contact frequency (>0.8) with CoA in both simulations. Residues R278 and K288 also appear frequently within 3 Å of CoA in simulations of the 8E50 system, while simulations of the AF+ligands system find that R278 and R461 frequently appear around CoA; see Figure 6d for the contact frequencies of the selected residues. Notably, R278 is located on motif II, which is known for binding with CoA. Other residues on motif II also interact with CoA frequently: G273, I276 and R277. Their contact frequencies and conformations can be found in Figure S12. While the results support the prediction of motif II’s function, we note that motif II alone is not sufficient to bind the entire CoA stably. The non-polar (hydrophobic) pocket to accommodate the acyl group and the positively charged residues at the pocket entrance to interact with the phosphate groups are still the key features for CoA’s binding.

**Figure 7:**
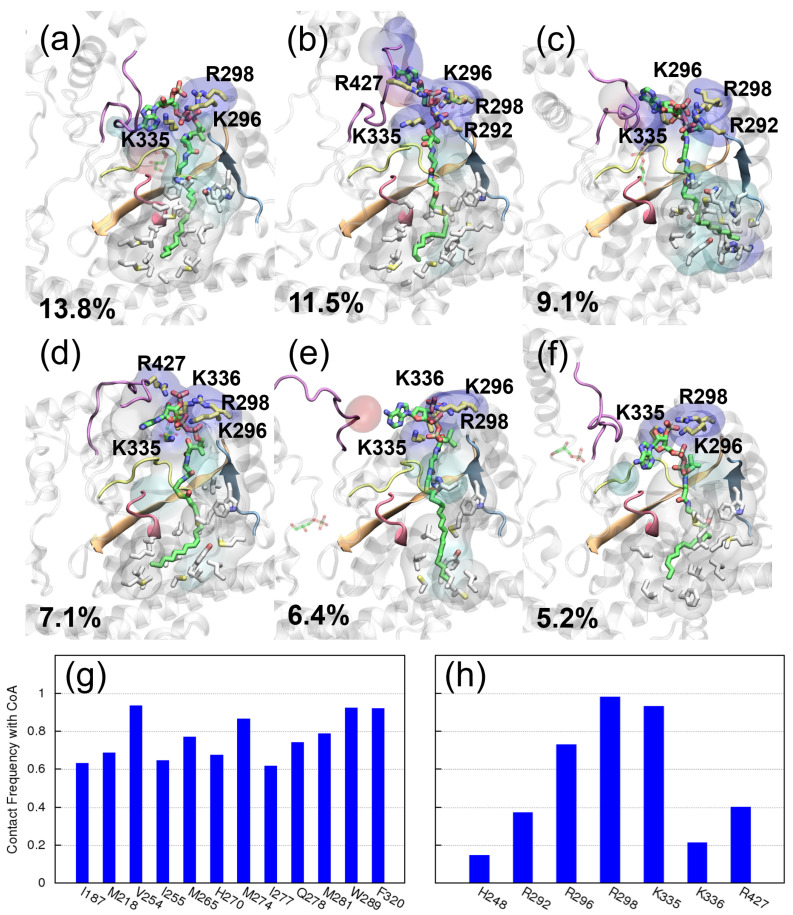
CoA’s binding with GPAT4. (**a**–**f**) Centroids of the first six clusters found in GPAT4’s MD simulations, respectively. As in Figure 6, positively charged residues within 3 Å of CoA’s phosphate groups are colored in yellow, residues within 3 Å of CoA’s acyl group are colored in white and the transparent volume depicts all protein residues within 3 Å of CoA. The color of the volume reflects the properties of the residues: positively charged (blue), negatively charged (red), polar (cyan) and hydrophobic (white). The associated cluster size is listed as the percentage of the total trajectory length. (**g**) The contact frequency of the residues within 3 Å of the acyl group. Depicted are only those with a frequency larger than 0.5. (**h**) The contact frequencies of selected residues with CoA. Selected are residues that can interact with CoA’s phosphate groups and the catalytic H248.

CoA’s binding with GPAT4 follows a similar pattern: the acyl group binds at the non-polar pocket, as depicted by the white residues in Figure 7a–f. In contrast to the GPAT1 case, here, this acyl group explores many different conformations, suggesting that GPAT4 has a larger or a more flexible pocket. This is also reflected in the contact frequencies of the residues with CoA. For the residues that appear within 3 Å of the acyl group, their frequencies are generally lower than those in GPAT1, cf. the frequencies depicted in Figure 6c and Figure 7g. On the other hand, CoA’s phosphate groups are anchored by multiple positively charged residues at the pocket entrance. Among these arginine and lysine residues, K296, R298 and K335 interact with CoA with a higher contact frequency (>0.7), while R292 on motif II and K336 interact with CoA less frequently, as revealed by the contact frequency statistics in panel (g). Interestingly, R427 sometimes can interact with CoA and G3P simultaneously; see and compare Figure 7b,d and Figure 5c,e. Residues P286, H287, W289 and F290 from motif II also interact with CoA. See Figure S13 for their contact frequencies as well as their conformations. Our MD results support the predicted function of motif II for both GPATs, although the sequences are less conserved over GPAT1 and GPAT4.

Overall, our simulations support the function of motif II. However, acyl–CoA is too large to be stably bound by a single motif. The binding of CoA requires the combination of a deep non-polar pocket to accommodate the hydrophobic acyl group, as well as several positively charged residues at the pocket entrance to attract the phosphate groups.

## 3. Materials and Methods

The sequence alignment is performed using Clustal Omega [18,26]. Sequences are taken from UniProt [27] with access codes Q9HCL2, Q6NUI2, Q53EU6, Q86UL3, P0A7A7, Q4QMF0, Q61586 and P97564 for human GPAT1, human GPAT2, human GPAT3, human GPAT4, *Escherichia coli* GPAT, *Haemophilus influenzae* GPAT, mouse GPAT1 and rat GPAT1, respectively.

The molecular docking calculations are performed using smina [28], a fork of Autodock Vina that is designed for the easy customization of scoring functions and virtual screening. Prior to docking, the protein and ligands are first prepared: polar hydrogens are added to the protein with PyMol [29], and the ligand protonation state is determined using Open Babel [30]. CoA is first docked into the binding pocket using a search box defined by the CoA structure modeled from 8E50 and 4 Å padding in each direction. The docking exhaustiveness level is chosen to be 64, and the docking pose with the best docking score is taken. Next, G3P is docked to the binding pocket, using a search box based on the G3P structure taken from 8E50 and padding of 2 Å. This time, the exhaustiveness is chosen to be 16, which is sufficient to dock a small ligand such as G3P back to its crystal structure if available. The best docking pose is taken as the final (docking-constructed) complex structure. In other words, we rely on the docking software to find the binding pose based on the best docking score. As the docking performance strongly depends on the receptor’s conformation, e.g., the orientation of side chains, varying performance has been reported for docking with AlphaFold structures [21,22].

The MD simulations are performed using Gromacs [31]. Missing residues in 8E50 are modeled using Modeller [14]. The HNQ flip is predicted by reduce [32], and the protonation state is determined using propKa [33,34]. Since the two proteins are mostly exposed to the solvent, the composition of the membrane has a limited effect on the ligand binding. Thus, a simple membrane composed of 9:1 DOPC:cholesterol is constructed using CHARMM-GUI [23,35,36]. As a reference, we also construct systems of GPAT1 with a mitochondrial membrane (37% DOPC + 31% DOPE + 22% cardiolipin + 6% POPI) [37] as well as GPAT4 with an endoplasmic reticulum membrane (54% DOPC + 20% DOPE + 11% POPI + 8% cholesterol) [37]. The membrane composition does not affect how GPAT1 and GPAT4 are integrated onto the membrane; see Figure S14. Consequently, the membrane composition does not affect the simulation results either, if the simulation is sufficiently long; see Figures S15–S17 for the results obtained using the more sophisticated membranes. The orientation of GPAT relative to the membrane is predicted by the PPM Web Server [38], which takes the 3D structure as input to calculate the rotational and translational position of a transmembrane or peripheral protein. The pH value is set to 7. The system is then further solvated in 0.15 M NaCl solution, with a box size of 12.3 × 12.3 × 17.9 Å^3^. The CHARMM36m [39] and CGenFF [40] force fields are utilized for the simulations. Prior to the production runs, the system is subjected to 5000-step energy minimization, followed by 1 ns NVT equilibration and 2 ns NPT equilibration, where the restraints on the complex and lipids are gradually released. For the MD simulations, an integration step size of 2 fs is employed. The Verlet list is updated every 20 steps. The van der Waals interaction is gradually switched off at the internuclear distance range between 10 Å and 12 Å, while the electrostatic interaction is treated by Particle-Mesh-Ewald summation [41,42] with a cutoff at 12 Å and grid spacing of 1.2 Å. The temperature and pressure are controlled at 303.15 K and 1.0 bar by a Nosé–Hoover thermostat and a Parrinello–Rahman barostat (semi-isotropic), respectively. Bonds with hydrogens are restrained using the LINCS algorithm [43]. Finally, multiple copies of 500 ns MD simulations are executed, and the trajectories are combined for analysis using the Gromos clustering algorithm [44] as well as in-house VMD scripts for other analyses. Figures and movies are created with VMD [20].

## 4. Conclusions

GPAT catalyzes the conversion of G3P and CoA to LPA. As this conversion is the first and the rate-limiting step in TAG synthesis, GPAT becomes a therapeutic target to treat TAG-regulation-related diseases like obesity, hepatic steatosis and insulin resistance. To effectively design inhibitors to target GPAT and treat these diseases, the detailed binding mechanisms and important residues involved in recognizing G3P should be determined. In this work, we used MD simulations to identify the binding mechanism and important residues involved in the GPAT4–G3P–CoA complex. Normally, computational binding mechanism study requires experimentally resolved structures to obtain reliable results. However, there is no available experimentally resolved GPAT4 structure yet. Hence, we first conducted a comparative study on the GPAT1–G3P–CoA system to prove the feasibility of using AlphaFold models with well-addressed binding poses to reproduce the most important interactions observed in MD simulations on experimentally resolved structures. Then, we conducted MD simulations on the AlphaFold GPAT4 model with the ligand binding poses taken from the experimentally resolved GPAT1–G3P–CoA complex to identify the substrate recognition mechanism and the crucial residues involved in the GPAT4–G3P–CoA system. Such an operation was based on the assumption that GPAT1’s and GPAT4’s binding pockets were sufficiently similar, which was supported by the protein structures, which were predicted with high confidence. Our simulations revealed that residue R427 is responsible for recognizing G3P, but it is located on an extremely flexible region. Consequently, its motion brings G3P to various binding hotspots, formed by different positively charged residues. In the current study, five binding modes were identified based on the internuclear distance between G3P and selected residues. Transitions between these binding modes were also observed during the MD simulations. Our simulations also support the proposed function of motif II. However, a few residues are not sufficient to bind CoA stably. Rather, a non-polar (hydrophobic) binding pocket and multiple arginines or lysines are required to anchor CoA’s acyl group and CoA’s phosphate groups, respectively.

In this work, we propose a method for the computational modeling of a protein–substrate complex using the AlphaFold model together with substrates from the experimentally resolved structure of a homolog. Hopefully, the work also provides insights for future structure-based drug design targeting GPAT4 to treat various diseases. Although the results of our simulations are still limited by the accuracy of the structural prediction as well as the affordable simulation length, we hope that this study can stimulate more experiments to verify how G3P binds with GPAT4, e.g., site mutation to verify R427’s role in ligand binding.

## Figures and Tables

**Table 1:** Conditions to identify G3P binding modes. The distance is measured from the phosphorus atom on G3P to the selected atom on a residue (NZ for lysine, CZ for arginine, NE for histidine). All distance cutoffs are set to 5 Å, except for H248-G3P, where the cutoff is set to 11 Å. These values are chosen according to the steady interatomic distances observed in Figure S10. Mode 6 is a dummy used to gather all binding modes that do not belong to modes 1 to 5. The size of each mode in terms of the percentage of the trajectories is also listed.

Mode ID	Conditions	Size (%)
1	R427-G3P, H248-G3P	32.3
2	K296-G3P, R292-G3P	17.8
3	R427-G3P, K426-G3P	9.1
4	R148-G3P, R374-G3P	8.9
5	R427-G3P, K365-G3P	13.2
6	all and not above	18.7

## Data Availability

The trajectories and analysis scripts are available upon request from the corresponding author.

## Supplementary Materials

The following supporting information can be downloaded at: https://www.mdpi.com/article/10.3390/ijms25073729/s1.
